# Catalytically active and thermally stable core–shell gold–silica nanorods for CO oxidation†

**DOI:** 10.1039/d1ra01577j

**Published:** 2021-03-22

**Authors:** Yidong Chen, Sarah Lerch, Zafer Say, Christopher Tiburski, Christoph Langhammer, Kasper Moth-Poulsen

**Affiliations:** Department of Chemistry and Chemical Engineering, Chalmers University of Technology SE-412-96 Gothenburg Sweden kasper.moth-poulsen@chalmers.se; Department of Physics, Chalmers University of Technology SE-412-96 Gothenburg Sweden clangham@chalmers.se

## Abstract

Deactivation based on sintering phenomena is one of the most costly issues for the industrial application of metal nanoparticle catalysts. To address this drawback, mesoporous silica encapsulation is reported as a promising strategy to stabilize metallic nanoparticles towards use in high temperature catalytic applications. These protective shells provide significant structural support to the nanoparticles, while the mesoporosity allows for efficient transport of the reactants to the catalytically active surface of the metallic nanoparticle in the core. Here, we extend the use of gold nanorods with mesoporous silica shells by investigating their stability in the CO oxidation reaction as an example of high temperature gas phase catalysis. Gold nanorods were chosen as the model system due to the availability of a simple, high yield synthesis method for both the metallic nanorods and the mesoporous silica shells. We demonstrate the catalytic activity of gold nanorods with mesoporous silica shells at temperatures up to 350 °C over several cycles, as well as the thermal stability up to 500 °C, and compare these results to surfactant-stabilized gold nanorods of similar size, which degrade, and lose most of their catalytic activity, before reaching 150 °C. These results show that the gold nanorods protected by the mesoporous silica shells have a significantly higher thermal stability than surfactant-stabilized gold nanorods and that the mesoporous silica shell allows for stable catalytic activity with little degradation at high temperatures.

## Abstract 2

Gold nanorods stabilized with mesoporous silica shells are characterized and used as the catalyst for a model CO oxidation reaction to counter the costly sintering phenomena found in many industrial nanoparticle catalysts.

## Graphical abstract

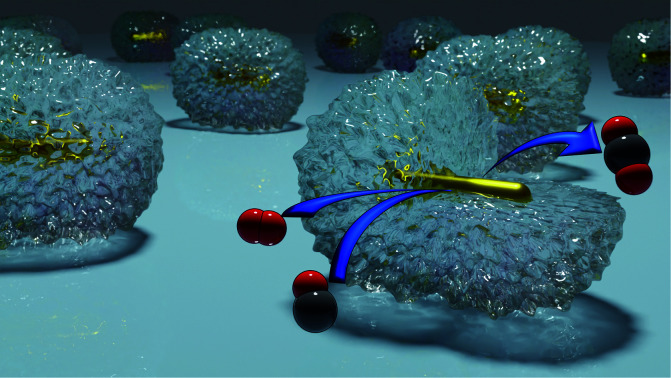

## Introduction

Metallic nanoparticles (NPs), particularly noble metals, like platinum (Pt), palladium (Pd) and gold (Au), are of great importance in the chemical industry, and in the fields of biomedical engineering, sensing and catalysis.^1–3^ Especially within the field of catalysis, the remarkable advances in the synthesis of shaped NPs signifies that their applications are steadily diversifying.^4–6^ When these shaped NPs are used as catalysts, it is observed that their overall catalytic performance can widely vary, depending on the crystal structure and specific facets. Recent experiments and computational simulations conclude that for a variety of structurally-sensitive catalytic reactions, the activity and selectivity can be improved by manipulating the arrangement of atoms on the surface.^7^ Therefore, different catalytic activity and selectivity may be achieved by engineering NPs with different crystal facets, as elegantly enabled by colloidal synthesis.^8^

When applying NPs as heterogeneous catalysts, it is important to consider the ultimate goal of providing efficient catalysis on an industrial scale. However, many industrial catalytic applications are normally performed above 300 °C,^9–11^ where the organic capping agents used as stabilizers for the NPs will decompose. In addition, due to their small size and high surface area, NPs are prone to sinter and coalesce when subjected to high temperatures.^12^ Therefore, it is anticipated that the highly active facets of shape-selected nanocrystals will gradually be destroyed at elevated temperature. Specifically, first the capping agents used during synthesis will thermally decompose, followed by recrystallization into the thermodynamically stable Wulff-shape structures and/or sintering into larger superstructures. Currently, in industrial applications, most heterogeneous catalytic processes take place on small spherical NPs, or NPs of undetermined shapes and non-uniform distributions because of their large surface area.^13^ Moreover, at elevated temperatures, catalytic processes on bare NPs usually take place on NPs adsorbed or grown on support materials, such as alumina,^14^ titanium oxide,^15^ and porous carbon,^16^ to maximize NP dispersion and improve their structural stability at elevated temperatures. As a consequence of the described intrinsic thermal instability of NPs with a well-defined shape, when used in catalysis, their application is usually restricted to mild thermal conditions and/or limited reproducibility. For instance, in Bai's work,^17^ bare Au nanorods (NRs) were tested for the reduction of nitro compounds, performed at ambient temperature, while Park, *et al.* reported a catalytic test of bare Pt NPs for CO oxidation up to 240 °C.^18^ Simultaneously, recent applications of unsupported catalysts indicate that the traditional need for a support to enhance the catalytic activity of NPs may be unnecessary when the NPs are optimized to the reaction.^19,20^

In a quest to overcome the thermal instability of NPs with well defined shapes, several recent publications have demonstrated that the use of the metal/mesoporous silica core–shell NPs, which can be achieved through several synthetic methods, are of great promise.^21,22^ The first method used to synthesize such structures, also called the surface permeable etching method, begins with the addition of silica monomers to a colloidal solution of pre-synthesized, facetted NPs and obtains porosity through the subsequent mesoporogen of these metal–silica core–shell structures.^23^ A second method also begins with a colloidal NP solution, but the porous structure is achieved by mixing the silica precursor and suitable amount of surfactants simultaneously.^24^ The porous coating provided by both of these methods allows for NPs with both shape stability and reactant penetration through the shell, allowing the reactants to interact with the active surface on the NPs during reactions. Recently, Zhang, *et al.* demonstrated that ultrasmall Au nanospheres encapsulated by porous silica exhibited stable activity for CO oxidation at high temperatures without compromising the original shape and size.^25^ Additionally, TTAB-stabilized Pt nanocubes with mesoporous silica shells produced inspiring, thermally stable catalysis for CO oxidation, with a stable catalytic performance recorded at 750 °C.^26^ In fact, in many cases, CO oxidation serves as a prototypical reaction for heterogeneous catalysts, including Au. While the mechanism of the catalysis varies depending on the particle orientation, particle size, particle shape and presence of a support oxide material,^15,16,19,22,27^ typically the heterogeneous catalysts serve to convert CO to CO_2_, since CO is a toxic gas, while CO_2_ is identified as an energy carrier for the eventual formation of renewable energy.^28,29^ Thus, the CO oxidation reaction is both industrially relevant and vitally important to environmental cleanup.

Au NRs are less frequently reported in catalytic applications than spherical Au NPs due to low temperature sintering and aggregation, as well as the presence of the surfactant, hexadecyltrimethylammonium bromide (CTAB), that isolates the active Au surface from external reactants.^5,30^ Therefore, the surface of Au NRs needs to be tailored for thermal stability as well as increased catalytic activity through the restructuring of the surface atoms and removal of CTAB.^31^ Recent examples include the coating of Au NRs with silica for the reduction of 4-nitrophenol^30^ and the use CTAB-capped Au nanorods functionalized by ionic liquid termination for the catalysis of nitro-compound reductions.^17^ However, as these reactions can occur at relatively low temperatures (<60 °C), the surface reconstructions were primarily highlighted for the increased catalytic activity, rather the high-temperature stability that is needed for a variety of other reactions, including CO oxidation.

Herein, we extend the use of Au NRs@mSiO_2_ for gas phase CO oxidation, which requires higher temperatures than the aforementioned solution based nitro-compound reductions. This reaction allowed us to highlight the effect of the mSiO_2_ coating, resulting in an enhanced thermal stability and improved catalytic performance of CTAB-stabilized Au NRs. Specifically, we synthesize Au NRs with high shape purity, and the corresponding Au NRs@mSiO_2_, using the surface permeable etching method. Subsequently, we compare the thermal stability, CO oxidation performance and durability of Au NRs and Au NRs@mSiO_2_ when catalyzing a high temperature oxidative/reductive reaction. As the main results, we find that the Au NRs@mSiO_2_ demonstrate steady activity and only minor degradation after several heating and catalytic treatments, as a consequence of their high thermal stability confirmed using electron microscopy.

## Experimental

All chemicals were obtained from the commercial suppliers as mentioned below and used without further purification. Milli-Q water (Milli-Q Advantage A10 Water Purification, Merck) was used for all solution preparation. All glassware and magnetic bars were cleaned using freshly prepared aqua regia (6 : 1 v/v HCl : HNO_3_), followed by rinsing with copious amounts of Milli-Q water.

### Chemicals

Chemicals were obtained from Sigma Aldrich, including hexadecyltrimethylammonium bromide (CTAB, ≥98%), hydrogen tetrachloroaurate trihydrate (HAuCl_4_·3H_2_O), l-ascorbic acid, silver nitrate (AgNO_3_), sodium borohydride (NaBH_4_), hydrochloric acid (HCl, 37 wt% in water), 3-mercaptopropyltrimethoxysilane (MPTMS), sodium silicate solution, tetraethyl orthosilicate (TEOS), sodium hydroxide (NaOH), and potassium iodide (KI). Other chemicals obtained from additional suppliers were sodium oleate (NaOL, TCI), nitric acid (HNO_3_, 70%, Merck), ethanol (analytical pure, Solveco) and iodine (I_2_, VWR Chemicals).

### Characterization

A Heraeus Multifuge X1 with a Fiberlite F15-8 × 50cy Fixed Angle Rotor was used for all centrifugation. Transmission electron microscope (TEM) images were obtained with a FEI Tecnai T20 operated at 80 and 200 kV. TEM samples were prepared by drop-casting NP suspensions on copper TEM grids with carbon membranes (01840-F, PELCO). Samples for TEM analysis before and after the catalytic tests were drop-cast on specially designed TEM windows composed of a silicon frame covered by a thin layer silicon nitride, which can survive the high temperatures needed for the catalytic tests. Zeta-potential was measured with a Malvern Panalytical Zetasizer Nano ZS. UV-Vis absorption spectra were measured using a temperature-controlled (27 °C) Agilent Cary-60 spectrophotometer with a xenon ash lamp (80 Hz) as a light source. Catalytic tests were performed using a Insplorion X1 (Insplorion AB, Gothenburg Sweden) connected with a PrismaPlus QME 220 mass spectrometer to measure the reaction output.

### Synthesis of Au nanorods

Ye *et al.*'s synthesis method^32^ was followed for high yield synthesis of Au NRs. First, a Au seed solution is prepared by reducing a mixture of HAuCl_4_ (0.5 mM, 5 mL) and CTAB (200 mM, 5 mL) with NaBH_4_ (6 mM, 1 mL) while stirring vigorously (1200 rpm) for 2 minutes. The brown seed solution was then aged for 30 minutes prior to growth. The growth solution consisted of a mixture of CTAB (7 g) and NaOL (1.234 g) dissolved in warm water (50 °C, 250 mL), which was cooled to 30 °C after the surfactant powders were completely dissolved. AgNO_3_ (4 mM, 18 mL) was added to this solution and aged for 15 minutes, followed by the addition of HAuCl_4_ (1 mM, 250 mL), which was stirred (700 rpm) for 90 minutes, until the solution became colorless. The pH of the solution was adjusted with HCl (12.1 M, 3 mL) and stirred (400 rpm) for a further 15 minutes, followed by the addition of ascorbic acid (64 mM, 1.25 mL) under vigorous stirring for 30 seconds. Finally, the seed solution (0.8 mL) was injected and stirred for 30 seconds before the solution was aged (without stirring) for 17.5 hours, resulting in an opaque, red-brown solution. The final products were isolated *via* centrifugation (7000 rpm, 30 minutes) and washed further, using both ultra centrifugal filters (MWCO 100 kDa, Aldrich) at 5100 rpm for 15 minutes and normal centrifugation (3200 rpm, 20 minutes). After washing, the highly concentrated NRs solution was carefully re-dispersed in Milli-Q water. The concentration of the NRs colloidal suspension was adjusted to 1 mM Au after the washing, to prepare for the silica coating. We have assumed that the amount of Au NRs remains invariant during the mesoporous silica coating.

### Synthesis of Au nanorods@mSiO_2_

The coating of a mesoporous silica shell is achieved by following Zhou's work^33^ with two modifications. First, the strictly washed Au NRs suspension from the previous sections is coated instead of applying the Au NPs from Zhou's protocol. Additionally, the centrifugation speed was changed to 3200 rpm to fit the size requirements of our Au NRs. Briefly, this method is a surface permeable etching method that begins with the modification of the Au NR (1 mM Au, 4 mL) surface achieved by stirring with MPTMS (0.54 mM, 150 μL) followed by the growth of a thin silica shell with sodium silicate (0.54% SiO_2_ in weight, pH 10–11, 150 μL). Subsequently, a thick silica shell is formed through the addition of TEOS (10 v%, in ethanol, 160 μL) and NaOH (0.1 M, 100 μL) and mesopores are obtained through an etching step using NaOH (0.1 M, 70 μL) and CTAB (0.1 M, 140 μL). A detailed synthetic diagram of this process is included in the ESI S1.1.†

### Core etching of Au nanorods@mSiO_2_

The core-etching protocol was adapted from the work of Chul Cho, *et al.*^34^ 1.5 mL I_2_/KI solution (0.34 mM I_2_, 2.04 mM KI) was introduced into the Au NRs@mSiO_2_ suspension (1 mM Au, 1 mL) at room temperature, while stirring at 500 rpm. After 3 minutes, the suspension was recollected by centrifugation (3200 rpm, 15 minutes). The sedimented NPs at the bottom of the centrifugal tube were dispersed into 0.3 mL Milli-Q water for TEM imaging. A parallel spectroscopic test was performed to show the full etching procedure, with the suspension transferred to a quartz cuvette after 5 minutes of reaction time.

### CO-oxidation reaction on Au nanorods

The catalytic performance of Au NRs and Au NRs@mSiO_2_ was examined by monitoring the CO-oxidation catalytic reaction. In this work, a plug-flow type reactor has been utilized and a ‘glass pocket’ was integrated inside the externally heated quartz tube as previously reported by our recent work^20^ and Fredriksson *et al.*^35,36^ This unique reactor design with minimum dead volume enhances the quadrupole mass spectrometer (QMS) ion current for reaction products from our unsupported nanoparticle catalysts. Total gas flow during the reaction was set to 100 mL min^−1^ through the quartz tube and only 1 mL min^−1^ portion was directed to pocket reactor. Au NRs and Au NRs@mSiO_2_ samples (5 mL, 6 mM Au each) were drop-cast on separate, pre-diced 2 cm^2^ silica wafers and these wafers were placed in a flow reactor (detailed experimental diagram in the ESI, S5.1†). This resulted in approximately 6 × 10^12^ NRs, or 5.9 mg of Au provided as a dispersed catalyst. Samples were pre-treated with 5.0% O_2_ for 4 hours and 2.0% H_2_ for 1 hour at 500 °C prior to the CO oxidation reaction test, in order to remove organic residue and stabilize the NP surface (S5.2†). The catalytic test was run over 5 hours with a mixture of 0.5% CO and 5.0% O_2_ in an Ar carrier gas with a 1 mL min^−1^ flow rate, corresponding to reduced CO emission strategies with excess oxygen concentration in industry and mobile sources.^37^ This catalytic test was repeated three times to demonstrate stability of the catalyst through multiple treatments.

## Results and Discussion

### Preparation of mesoporous silica-coated Au NRs

**Fig. 1:**
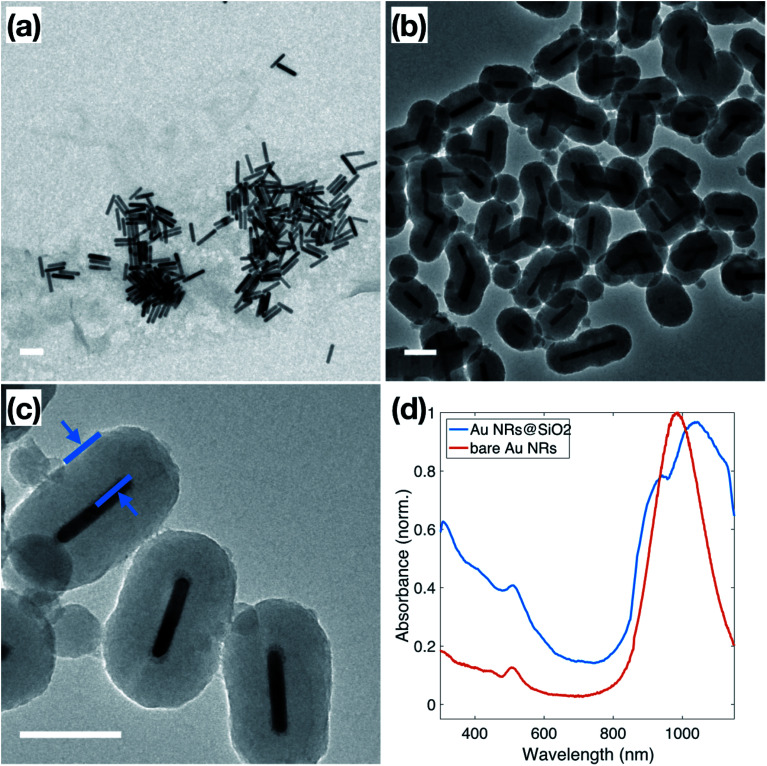
(a) TEM of surfactant-stabilized Au NRs approximately 14.1 ± 2.7 nm in diameter and 79.3 ± 7.3 nm in length; (b and c) TEM of Au NRs coated with SiO_2_ shell of approximately 50 nm. The distance indicated in blue in (c) is 48 nm. (d) UV-Vis spectra of surfactant-stabilized Au NRs (red) and Au NRs@SiO_2_ (blue). All scale bars represent 100 nm.

Au NRs were synthesized *via* a seed-mediated growth reaction, as described in the Experimental section, resulting in Au NRs with an average aspect ratio of 5.8 ± 1.8, shown in Fig. 1a. Based on TEM measurements, these Au NRs have dimensions of 14.1 ± 2.7 nm in diameter and 79.3 ± 7.3 nm in length. The as-synthesized solution of surfactant-stabilized Au NRs was washed three times, with normal centrifugation methods and ultra-centrifugal filters, respectively. The ultra-centrifugation was introduced to solve the formation of serious aggregations when sodium silicate is added to the NP solution, especially noticeable in solutions of spherical NPs.^38^ It is known that the condensation of silicate ions to form polymeric and colloidal silica is affected by the nature of the cations present in the solution. In Liz-Marzán's work,^39^ the NPs solution is pre-dialyzed in Milli-Q water for two days to remove excess ions before the silica coating. We chose to introduce the ultra-centrifugal filters to achieve the same goal. In particular, the ultra-centrifugal filters allow for the rapid removal of the excess surfactants without repeated washings, which often results in the aggregation of NPs, especially in CTAB-stabilized NPs solutions. Additionally, the removal of excess surfactant mixture allows for surface modification during the silica coating, while leaving enough surfactant on the Au NR surface to prevent aggregation. The zeta potential of the Au NRs solution, which dropped from 86.2 ± 2.5 mV to 24.0 ± 0.5 mV, was used to monitor NP stability throughout the washing steps, resulting in NPs that maintained enough charge for stability but with no excess ionic contributions from the solution.^40^

Normally, due to the lack of an –OH group, the surface of metallic NPs, stabilized only by the surfactant used in synthesis, does not have good affinity to SiO_2_, which is referred to as “vitreophobic”. Thus, before the formation of the silica shell, MPTMS, a bifunctional silane coupling agent, was applied to functionalize the NPs' surface. After the MPTMS functionalization, the silanol groups dangling outward from the NP surface enable the hydrolysis and condensation of sodium silicate. Initially, enough sodium silicate was introduced to grow a thin layer of silicate around the gold surface, which improves the stability of the NPs for the further thicker silica shell growth, which occurs in an ethanol/water (EtOH/H_2_O) solution over two days. The thicker silica shell was grown onto these modified Au NRs through the addition of TEOS to the Au NRs solution, followed by 24 hours of reaction, stirred at room temperature. TEM images of these Au NRs@SiO_2_, in Fig. 1b, show clearly that the majority of the Au NRs were individually and uniformly coated with a dense silica shell after this process. The texture of this coating appears smooth, and the thickness is around 48–50 nm, as shown in Fig. 1c. Additional TEM images are presented in the ESI, S2.1,† including images to demonstrate the time dependence of the silica growth (S3.1†). The UV-Vis spectra, for the Au NRs, shown in Fig. 1d, also indicates the growth of the silica shell due to the shift of the plasmon resonances associated with the NRs. The bare Au NRs (red) have plasmon resonance peaks at 510 nm and 982 nm, corresponding to the transverse and longitudinal plasmons. After growth of the dense silica coating, there is a significant red shift of the longitudinal peak to 1038 nm, and slight shift of the transverse peak to 515 nm, due to the changing of local refractive index in the surrounding medium.^41,42^ The longitudinal peak also features a shoulder at approximately 950 nm, resulting from the nanorods that did not get efficiently coated.

**Fig. 2:**
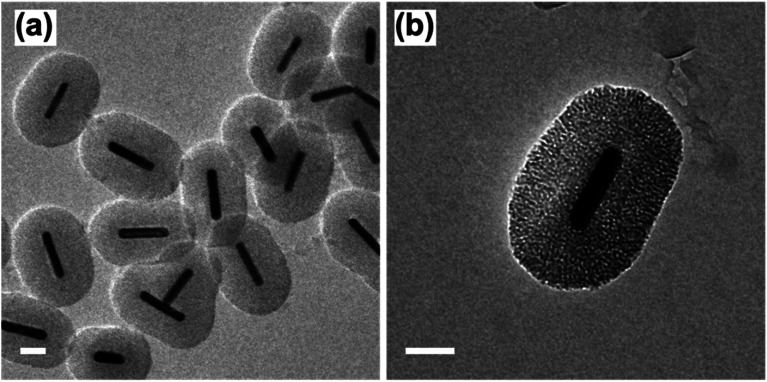
TEM images of Au NRs with mesoporous silica coating (Au NRs@mSiO_2_) before, (a) and after (b) 500 °C calcination treatment. All scale bars represent 50 nm.

Following the formation of the dense silica coating, the Au NRs@SiO_2_ were treated with NaOH, a typical mesoporogen, and additional CTAB in order to form the porous structure necessary for effective catalytic function. TEM images of these Au NRs@mSiO_2_ are shown in Fig. 2. The CTAB stabilizes the exposed Au NR surface as well as contributes to the formation of molecule-sized pores,^43^ but contaminates the porous structure, resulting in pores with inadequate resolution for imaging (Fig. 2a) and limits NP surface accessibility for catalysis. Thus, a 4 hour, 500 °C calcination treatment in air was applied to evaporate or decompose the CTAB that remained inside the pores. In Fig. 2c, pore structures are clearly visible after the calcination treatment. The thickness of this mesoporous silica coating is approximately 50 nm, which is equivalent to the former dense silica coating. Additional TEM images of Au NRs@mSiO_2_ are presented in the ESI, S2.2.†

### Thermal stability test

**Fig. 3:**
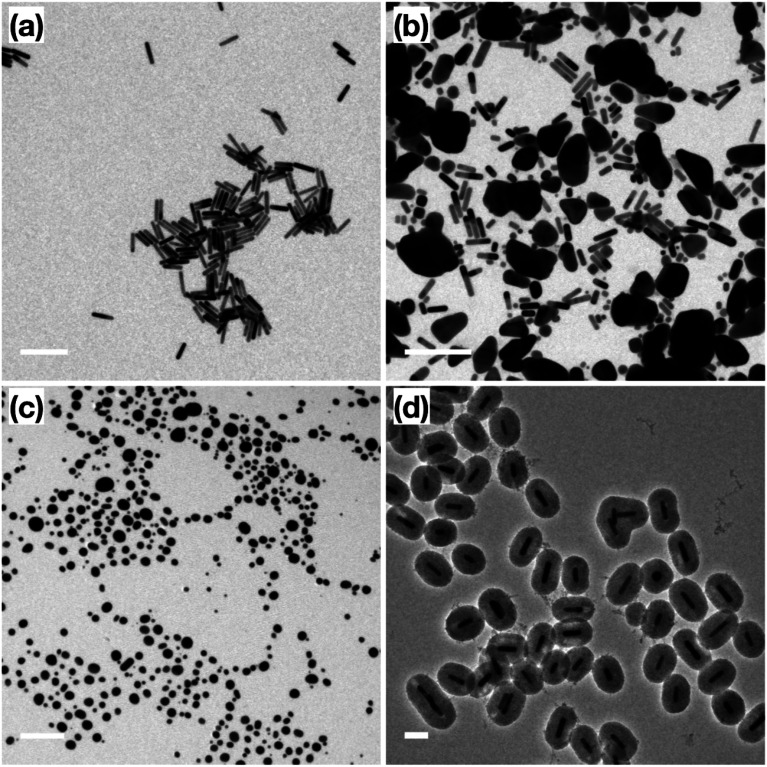
TEM images of (a) original surfactant-stabilized Au NRs; (b) surfactant-stabilized Au NRs after 150 °C treatment; (c) surfactant-stabilized Au NRs after 500 °C treatment; (d) Au NRs@mSiO_2_ after 500 °C treatment. Scale bar: (a and b) 200 nm, (c) 500 nm, (d) 100 nm.

Prior to catalytic application, a thermal stability test was performed on the surfactant-stabilized Au NRs, and Au NRs@mSiO_2_ to compare the impact of surface reconstruction due to the heat treatment, without potential catalytic deformation. The surfactant-stabilized Au NRs, dispersed and dried on TEM grids (Fig. 3a), were subjected to two temperature treatments, in order to determine the effect of temperature on the sample. The first treatment, at 150 °C (18 hours), resulted in the melting of a large portion of the Au NRs in the sample, predominately into micron-sized Au aggregates (Fig. 3b). The second treatment, designed to mimic the calcination process described above, included a gradual temperature increase from 25 °C to 500 °C over a 2 h period, followed by a stable temperature of 500 °C for 4 hours. Analysis of the sample following this heat treatment shows that all the Au NRs have sintered into irregularly sized Au spheres (Fig. 3c), clearly demonstrating the lack of thermal stability provided by the organic surfactants to these Au NRs. When compared to the post-calcination Au NRs@mSiO_2_ (Fig. 3d), where the rod shape is clearly maintained, it is clear that the mesoporous silica coating successfully improves the thermal resistance of Au NRs.

### Accessibility of Au surface to reactants

**Fig. 4:**
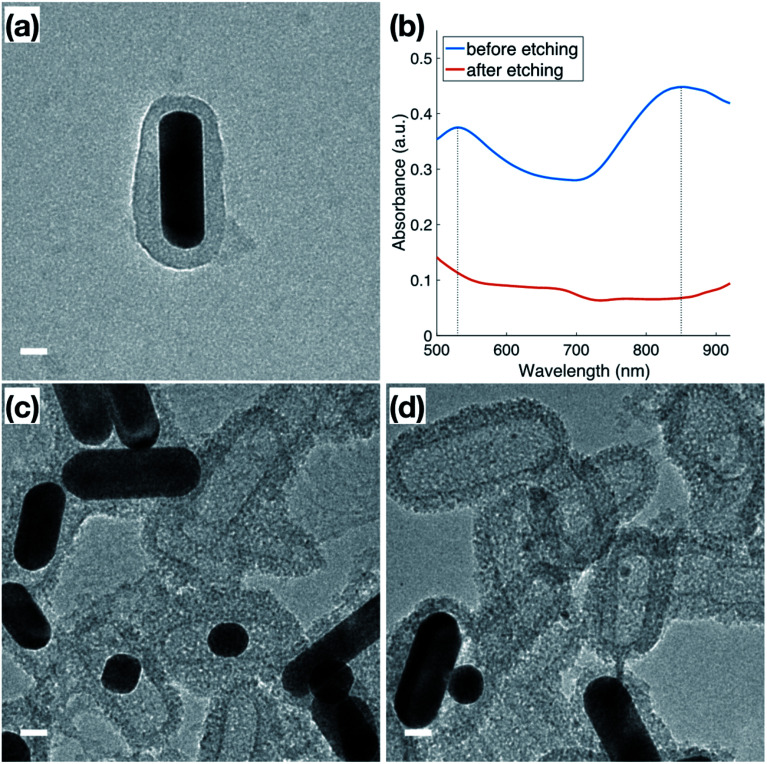
TEM images of (a) original Au NRs@mSiO_2_ after synthesis; (b) UV-Vis spectra of original Au NRs@mSiO_2_ (blue) and the Au NRs@mSiO_2_ after 5 min iodine etching (red). (c and d) Au NRs@mSiO_2_ during iodine etching. All scale bars represent 20 nm.

In addition to the thermal stability, the accessibility of the Au surface through the mesoporous shell before calcination (Fig. 4a) was investigated by etching the inner Au core with I_2_/KI, where I_2_ is the main etchant and the KI is added to improve solubility. The Au NRs used for this etching featured a slight variation in dimensions from those used for catalysis, indicated by the plasmon peaks observed at 530 nm and 850 nm (Fig. 4b) in the original Au NRs@mSiO_2_ solution. Following 5 minutes of the I_2_/KI etching procedure, there is a clear indication that all the Au NRs were etched through the shell as both plasmonic peaks vanish. Additional spectroscopies with a same concentration of I_2_/KI demonstrating the time-lapse of the etching procedure and another spectroscopy using lower concentration of I_2_/KI are also included in the ESI (Fig. S4.1 and S4.2, respectively).† The TEM images in Fig. 4c and d capture the vivid changes at approximately 3 minutes into the etching process. Au NRs are missing, or are etched into smaller spheres, in a majority of the sample, leaving hollow, mesoporous silica shells, demonstrating that the inner nanoparticle cores are still accessible to small molecules in the outside environment, even before the CTAB is fully removed from the mesopores *via* calcination. This demonstrates that the mesoporous silica shell improves the thermal stability of these Au NRs, but does not block the movement of reactants to the inside, metallic core. Accessibility of Au NR in the core was further confirmed through reactivity towards gas phase CO oxidation reaction at high temperature, as will be discussed in next section in detail.

### Catalytic performance

The catalytic performance of bare Au NRs and the corresponding Au NRs@mSiO_2_ was evaluated by using high temperature CO oxidation as a model reaction.^44^

**Fig. 5:**
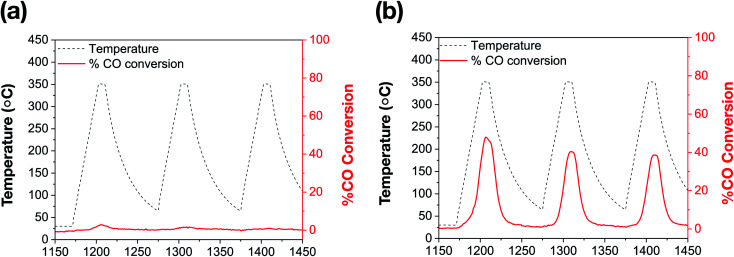
Catalytic thermal % CO conversion of (a) bare Au NRs; (b) Au NRs@mSiO_2_, following a 500 °C pre-treatment (ESI, S5.2†).

Surfactant-stabilized Au NRs were initially tested following a low temperature (150 °C) pre-treatment, and show no discernible CO conversion activity, which can be attributed to the inaccessibility of the Au surface due to the surfactant, CTAB, as well as the formation of larger Au domains corresponding to aggregation (Fig. 3b).^5,30,45^ As indicated by the aforementioned TEM images, the CTAB present on the surface decomposes and the ‘bare’ Au NRs lose their rod-like shape beyond 150 °C. Further catalytic treatments, performed up to 350 °C, exhibit expectedly low CO oxidation activity on these ‘bare’ Au NRs (Fig. 5a). However, Au NRs@mSiO_2_ demonstrate a significantly higher catalytic activity than the ‘bare’ Au NRs, and remain highly active up to 350 °C (Fig. 5b). This can be attributed to a reconstruction of the Au atoms upon silica coating, the removal of CTAB from the surface following the 500 °C pre-treatment (Fig. S5.2†) and the permeability of the mSiO_2_ surface to reactants. This limited catalytic activity (∼50% conversion) is governed by the total number of exposed accessible active sites of the Au NRs@mSiO_2_, which may be partially hindered by the dimensions of the porous structure of the coating. However, there is only a small degradation after several heating cycles, with the Au NRs@mSiO_2_ maintaining 95% of their initial activity after three cycles. The CO ion current calibration, as well as the CO_2_ production of bare Au NRs and Au NRs@mSiO_2_ in units of CO_2_ ion current are reported in the ESI S5.3 and S5.4,† respectively.

**Fig. 6:**
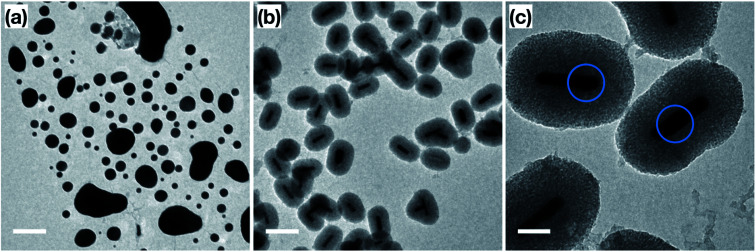
TEM images of (a) ‘bare’ Au NRs; (b and c) Au NRs@mSiO_2_ after the CO oxidation test. Scale bar: (a and b) 200 nm, (c) 50 nm. Blue circles are added to emphasize the deformation around the NR surface.

In order to confirm the thermal and catalytic stability of the Au NRs@mSiO_2_, we imaged the sample before and after the catalytic treatments. As demonstrated in Fig. 6a and b, the surfactant-stabilized Au NRs melt while the coated NRs clearly maintain their shape, size and the mesoporous silica shell. However, there are slight deformations, shown in Fig. 6c (circled), of the Au NRs inside of the mesoporous silica shell, which can be attributed to the reconstruction of the surface Au atoms as they reach more thermodynamically stable configuration during the initial pre-treatment process of the Au NRs (ESI, Fig. S5.5†) and during the high temperature catalytic reaction.

## Conclusion

In summary, we have synthesized Au NRs with an aspect ratio of 5.8 ± 1.8 and successfully coated these colloidal NRs with a ∼50 nm thick mesoporous silica shell. We characterized the thermal stability and surface accessibility of these Au NRs@mSiO_2_ compared to the surfactant-stabilized Au NRs. We have demonstrated and compared the catalytic activity, as well as the durability of both surfactant-stabilized Au NRs and Au NRs@mSiO_2_, respectively, using a CO oxidation model reaction with multiple heating cycles. Combined with the TEM results before and after these catalytic tests, we show that the increased catalytic activity and low degradation rate of the Au NRs@mSiO_2_ are attributed their excellent thermal stability and the removal of the surfactants at these high temperatures.

## Author contributions

YC synthesized and characterized the samples and contributed to the written manuscript. SL contributed to the characterization of the samples and wrote the manuscript. ZS and CT performed the catalytic experiments and contributed to the written manuscript. CL supervised the catalysis experiments. KMP supervised the nanoparticle synthesis and characterization. All authors have given approval to the final version of the manuscript.

## Conflicts of interest

The authors declare no competing financial interests.

## Supplementary Material

RA-011-D1RA01577J-s001
