# Supplementary material for: Catalytically active and thermally stable core–shell gold–silica nanorods for CO oxidation
Source: RSC Adv. 2021 Mar 22;11(19):11642–50. doi: 10.1039/d1ra01577j (PMC8695914; doi:10.1039/d1ra01577j)
Supplement: RA-011-D1RA01577J-s001 [file RA-011-D1RA01577J-s001.pdf]

Electronic Supplementary Material (ESI) for RSC Advances.
This journal is © The Royal Society of Chemistry 2021

Supporting Information

Catalytically active and thermally stable core-shell gold-silica nanorods for CO oxidation

Yidong Chen[a], Sarah Lerch[a], Zafer Say[b], Christopher Tiburski[b] Christoph Langhammer[b], Kasper Moth-Poulsen [a]*

[a] Department of Chemistry and Chemical Engineering, Chalmers University of Technology, SE-412-96 Gothenburg, Sweden

[b] Department of Physics, Chalmers University of Technology, SE-412-96 Gothenburg, Sweden.

Number of pages: 6
Number of figures: 9

Contents:

1. **Experimental supplement** 2
2. **TEM results of synthesized nanostructures** 2
3. **Discussion of the optimization on experimental duration** 3
4. **Iodine Etching** 4
5. **Catalytic characterization** 5

1. Experimental supplement

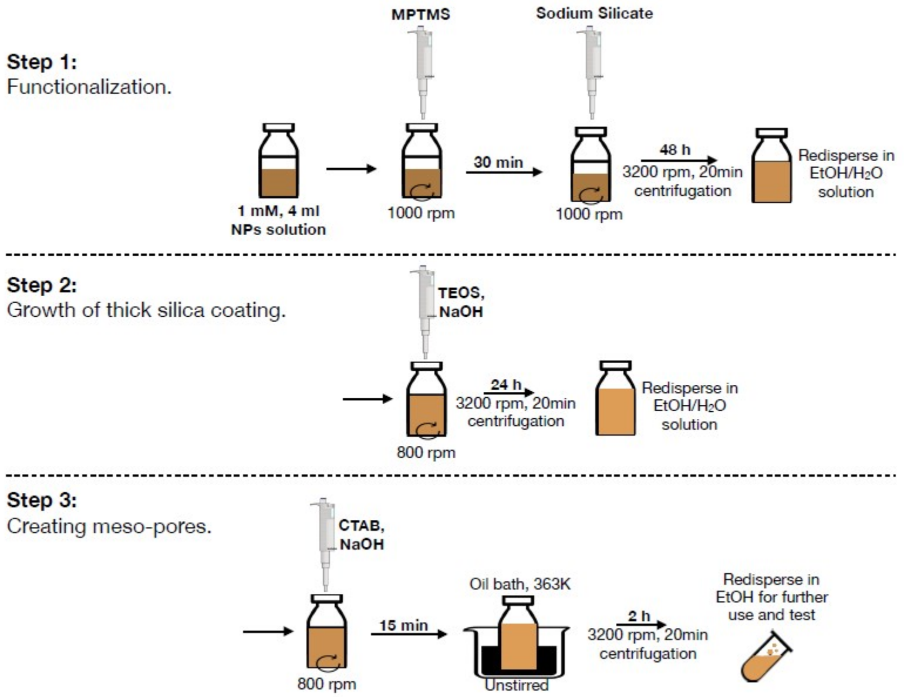

Figure S1.1. Experimental flow diagram of mesoporous silica coating step.

2. TEM results of synthesized nanostructures

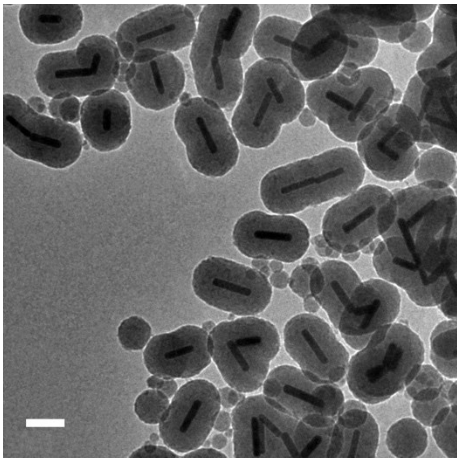

Figure S2.1. TEM of gold nanorods coated with SiO_2 shell of approximately 50 nm. Scale bar: 100 nm.

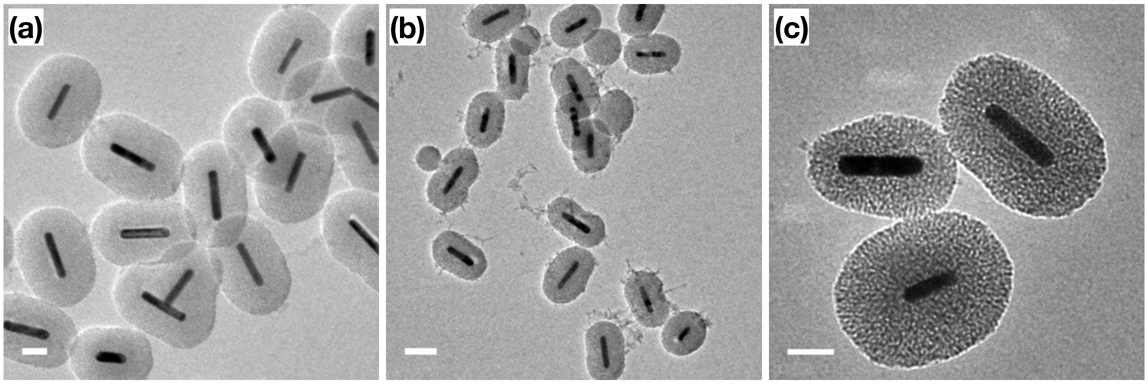

Figure S2.2. TEM images of (a) Au NRs with mesoporous silica coating (Au NRs@mSiO_2) before 500 °C calcination treatment; (b), (c) Au NRs@mSiO_2 after 500 °C calcination treatment. Scale bar: (a), (c) 50 nm, (b) 100 nm.

3. Discussion of the optimization on experimental duration

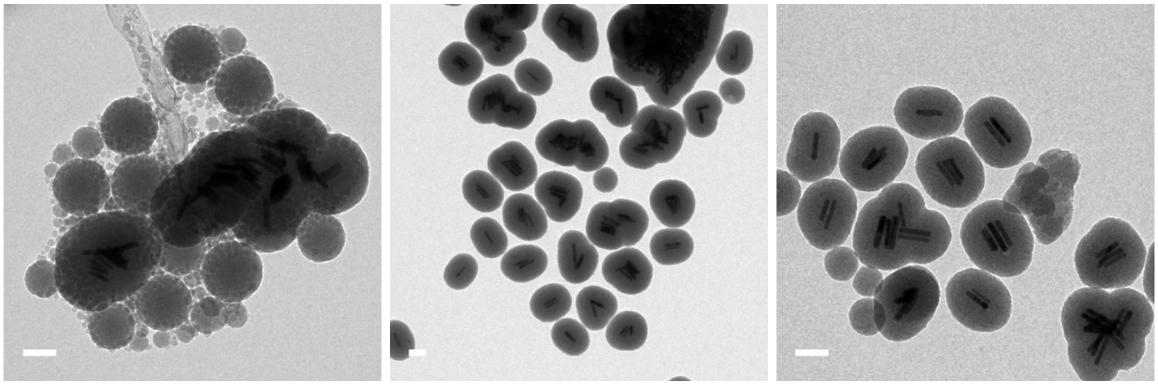

Figure S3.1. TEM pictures of Au NRs@SiO_2 after (a) 1 hour, (b) 6 hours, (c) 24 hours stirring time during the addition of sodium silicate. All scale bars represent 100 nm.

During the synthesis of Au NRs@mSiO_2, the experimental duration is significantly prolonged due to the thin silica shell creation, which takes 48 hours. Thus, different stirring times are tested to confirm the minimum length of time necessary to effectively coat the Au NRs in the initial thin silica shell. After adding the sodium silicate solution, the suspension is collected by centrifugation after 1 hour, 6 hours, and 24 hours, respectively. After centrifugation, these three parallel samples are redispersed for subsequent growth step with the TEOS source, as in the original protocol. Finally, Au NRs@SiO_2 are identified and Figure S3.1 shows the difference between these three samples. Notably, the TEOS source fails to coat single NRs for all three samples, and aggregations of NRs are coated instead, resulting in AuNRs@SiO_2 with multiple NR cores (Figure S3.1a and

b). However, from Figure S3.1 a to c, it is clear that as the stirring time is increased, the number of NRs in each silica shell decreases to 2-3 Au cores, indicating that the 48 hours stirring time is crucial for the formation of a thin silica shell around each individual particle, allowing the TEOS monomer to precisely capture the isolated NRs. This shows that, at least for the CTAB-&NaOL-stabilized Au NRs, the experimental duration cannot be significantly shortened.

4. Iodine Etching

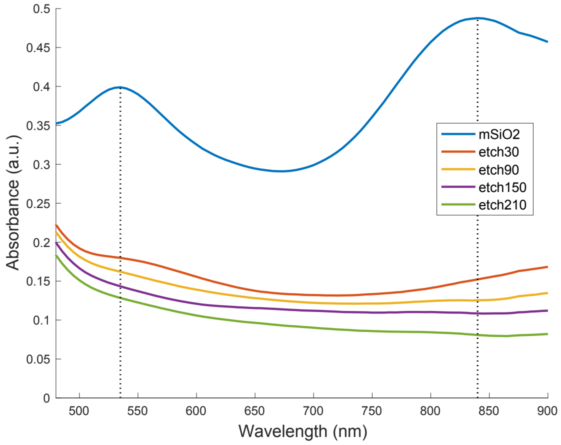

Figure S4.1. UV-Vis spectra of track-etched Au NRs@mSiO_2.

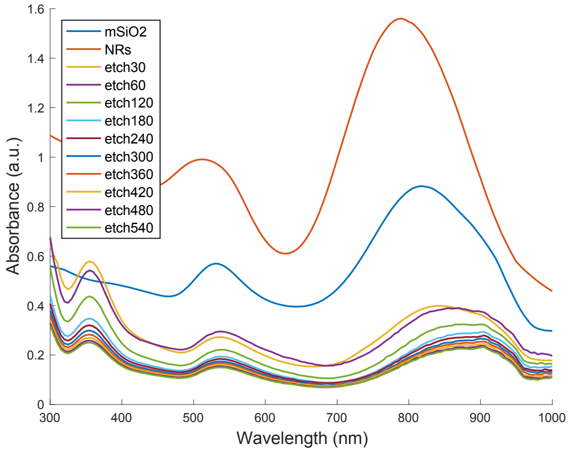

Figure S4.2. UV-Vis spectra of track-etched Au NRs@mSiO_2 with lower concentration of I_2/KI (0.075 mM).

Gold etching with lower iodine concentration was attempted to monitor the slower gradual etching process.

5. Catalytic characterization

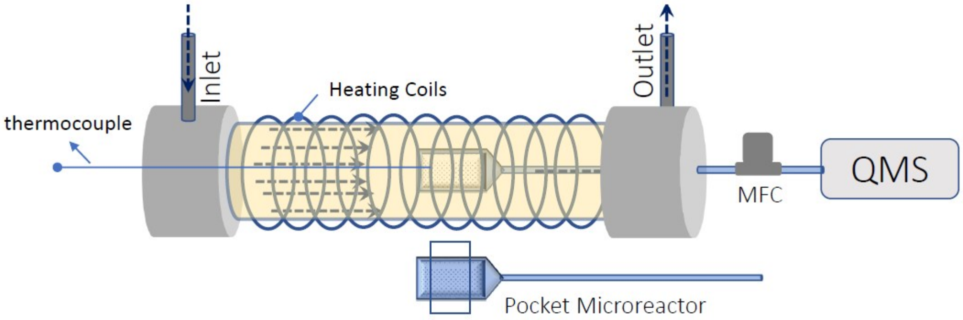

Figure S5.1. Schematic illustration experimental setup combining pocket reactor and Quadruple Mass Spectrometer (QMS).

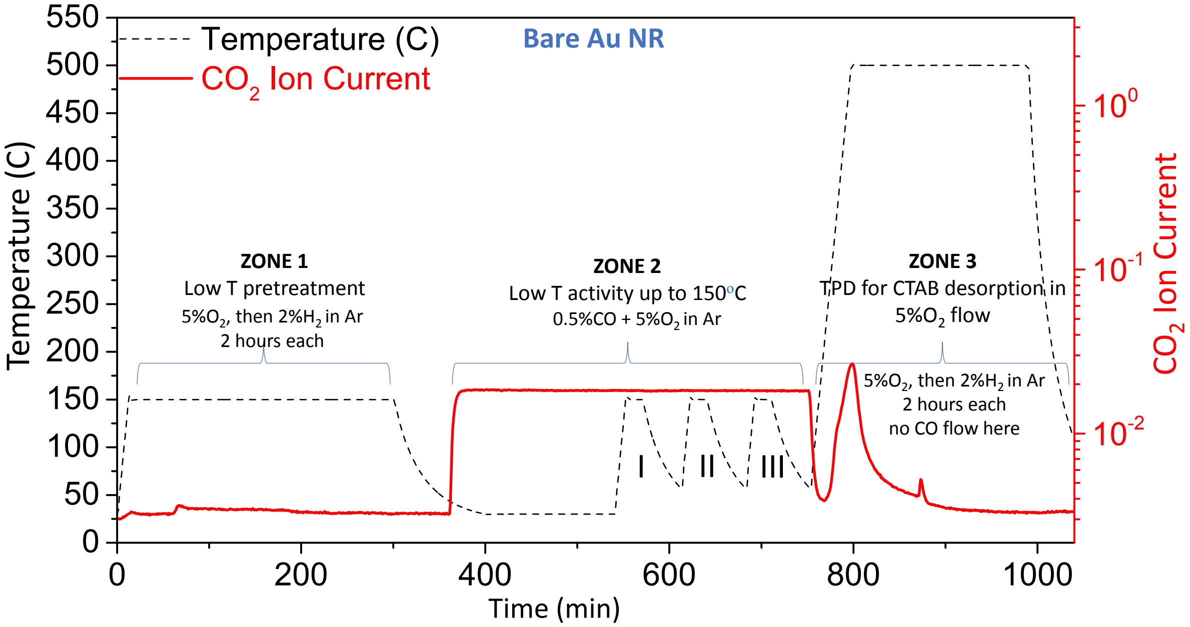

Figure S5.2. Schematic illustration pre-treatment experimental protocol.

In figure S5.2, we show the pre-treatment protocol that is applied to all samples prior to catalytic testing. Briefly, our catalytic experiments began with a low temperature (150°C) pre-treatment consisiting of 2 hours in 5% O_2 and 2 hours in 2% H_2, as show in zone 1. This was followed by the

introduction of a gas mixture of 0.5% CO and 5% O_2 in zone 2. It should be noted that the increase in the CO_2 ion current at the beginning of zone 2 is just a base line which is characteristic to our working base pressure of the mass spectrometer in the current reactant gas mixture and is, therefore, not related to CO oxidation. In zone 2, there are also three cycles (I, II, and III) of catalysis up to 150°C, where no CO_2 production was observed. This shows that the AuNRs are coated by organic surfactants so the catalysts is not yet active for CO oxidation. Therefore, in zone 3, we annealed the catalysts at high temperatures (500°C) in a 5% O_2 flow to remove the organics or other impurities from the surface of the catalyst. The organic materials, including CTAB, NaOL and MPTMS, decompose into CO_2 and N_2O, which have the same atomic mass (44 amu). This decomposition can be tracked by the increase of the ion current along with the linear heating ramp (10°C/min). Here, it is obvious that the CO_2/N_2O desorption, which originates from the decomposition of the organic surfactants, reaches a maximum during the temperature programmed desorption and completes around the 900th minute. This indicates that the final catalyst is free of organics/impurities and the Au surface is the active surface in the CO oxidation, as illustrated in the manuscript.

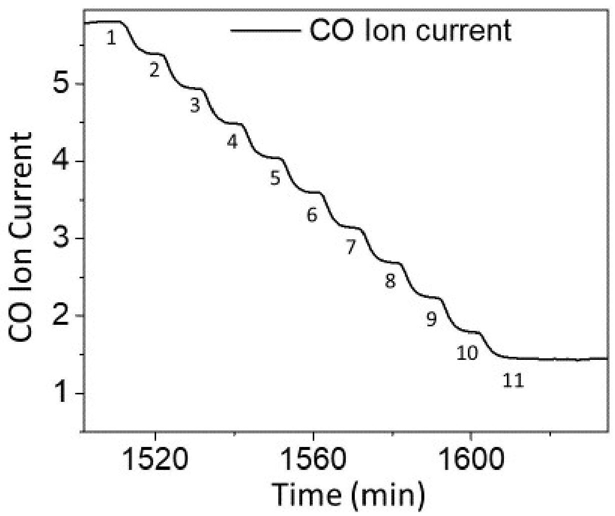

Figure S5.3. Corresponding calibration test for CO ion current at 11 different concentrations from 5000 ppm to 0 ppm. 1: 5000 ppm, 2: 4500 ppm, 3: 4000 ppm, 4: 3500 pm, 5: 3000 ppm, 6: 2500 ppm, 7: 2000 ppm, 8: 1500 ppm, 9: 1000 ppm, 10: 500 ppm, 11: 0 ppm.

In this work, the percent conversion performance, as reported in Figure 5, was calculated based on the CO consumption. We also include CO fragment build up from CO_2 production in our calculations. Finally, we integrated the results into the CO calibration, as shown in Figure S5.3. Our MA (Pfeiffer Thermostar) operates in default ionization potential, filament current and base pressure. We do no calibrate the spectrometer for each experiment, but we calibrate the ion current based on the different concentrations as shown here.

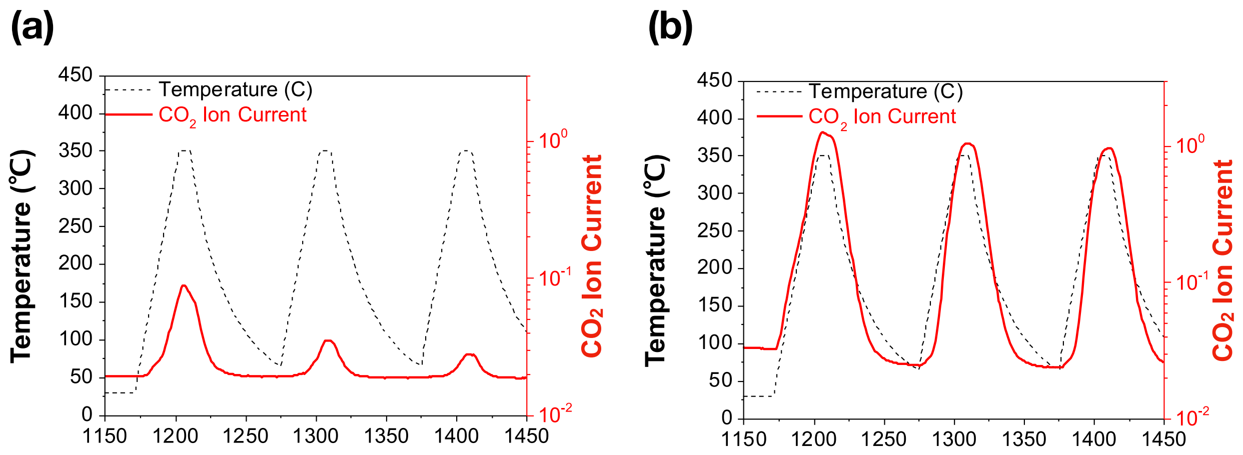

Figure S5.4. Catalytic CO_2 production activity of (a) bare Au NRs; (b) Au NRs@mSiO_2 in the unit of CO_2 ion current.

In figure S5.4, the data set exhibits the same trend as the catalytic results shown in the main article, but in the units of CO_2 ion conversion.

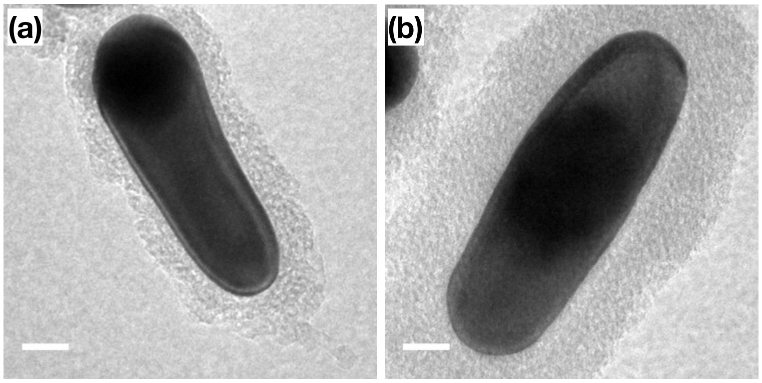

Figure S5.5. TEM pictures of Au NRs@mSiO_2 after the pre-treatment of the catalytic program. All scale bars represent 20 nm.

In order to further verify that if the shape deformations shown in the Figure 6c in the main article were formed during the pre-treatment of the catalytic test, the pre-treatment program was

performed on the Au NRs@mSiO_2 solely following by the further TEM test. In Figure S5.5, similar deformations were captured, which support our hypothesis in the main article.
